# MANAGEMENT OF PANCREATICOPLEURAL FISTULAS SECONDARY TO CHRONIC PANCREATITIS

**DOI:** 10.1590/0102-6720201700030014

**Published:** 2017

**Authors:** Everton CAZZO, Márcio APODACA-RUEDA, Martinho Antonio GESTIC, Fábio Henrique Mendonça CHAIM, Helena Paes de Almeida de SAITO, Murillo Pimentel UTRINI, Francisco CALLEJAS-NETO, Elinton Adami CHAIM

**Affiliations:** 1 Department of Surgery, Faculty of Medical Sciences, State University of Campinas;; 2 Faculty of Medicine, Pontifical Catholic University of Campinas;; 3 Department Internal Medicine, Faculty of Medical Sciences, State University of Campinas, Campinas, SP, Brazil

**Keywords:** Pancreatitis, chronic, Pancreatitis, alcoholic, Pleural effusion, Pancreaticojejunostomy, Fistula

## Abstract

**Introduction:**

Pancreaticopleural fistula is a rare complication of chronic pancreatitis.

**Objective:**

To describe pancreaticopleural fistula due to chronic pancreatitis and perform an extensive review of literature on this topic.

**Methods:**

Comprehensive narrative review through online research on the databases Medline and Lilacs for articles published over the last 20 years. There were 22 case reports and four case series selected.

**Results:**

The main indication for surgical treatment is the failure of clinical and/or endoscopic treatments. Surgery is based on internal pancreatic drainage, especially by means of pancreaticojejunostomy, and/or pancreatic resections.

**Conclusion:**

Pancreaticopleural fistula is a rare complication of chronic pancreatitis and the Frey procedure may be an appropriate therapeutic option in selected cases when clinical and endoscopic treatments are unsuccessful.

## RESUMO

**Introdução:**

A fístula pancreaticopleural é complicação rara da pancreatite crônica.

**Objetivo:**

Descrever a fístula pancreaticopleural consequente à pancreatite crônica e fazer revisão extensa da literatura sobre o tópico.

**Métodos:**

Revisão narrativa abrangente através de pesquisa online nas bases de dados Medline e Lilacs para artigos publicados nos últimos 20 anos.

**Resultados:**

Houve 22 relatos de casos e quatro séries de casos selecionadas. A principal indicação para o tratamento cirúrgico é a falha de tratamentos clínicos e/ou endoscópicos. A cirurgia é baseada na drenagem pancreática interna, especialmente por meio de pancreaticojejunostomias e/ou ressecções pancreáticas.

**Conclusão:**

A fístula pancreaticopleural é complicação rara da pancreatite crônica e o procedimento de Frey pode ser opção terapêutica apropriada em casos selecionados quando os tratamentos clínico e endoscópico não obtiverem êxito.

## INTRODUCTION

Chronic pancreatitis is a progressive and irreversible inflammatory process characterized by the replacement of the pancreatic parenchyma by fibrotic tissue. This disease has as main clinical manifestations chronic and incapacitating abdominal pain, and loss of the exocrine and endocrine functions of the pancreas. Patients frequently require endoscopic and/or surgical procedures for the treatment of disease-related complications^8^, being pancreaticopleural fistula very rare. It is estimated to occur in 0.4% of patients with pancreatitis, mostly resulting from chronic alcoholic pancreatitis^10^. It corresponds to a condition in which pancreatic secretions drain directly into the pleural cavity, resulting from a chronic inflammatory process, acute inflammation or traumatic or iatrogenic rupture of the pancreatic duct. Usually it presents as massive and relapsing pleural effusions, often on the left side and with high content of pancreatic amylase^34^. 

This study aims to describe pancreaticopleural fistulas caused by chronic pancreatitis and perform a review of the current literature on this topic.

## METHODS

A review of the literature published over the last 20 years was conducted through an online search for the MeSH terms “pancreatitis, chronic” AND “pleural effusion” AND “fistula” in Medline (via PubMed) and “pancreatitis, chronic OR pancreatite crônica OR pancreatitis crónica” AND “pleural effusion OR “derrame pleural” AND/OR “fístula” in Lilacs (via BVS). Original studies that reported single cases or case series of this disease or correlated conditions were included. Articles that consisted of *in vitro* or animal studies, articles in which the participants’ characteristics did not match those mentioned above, poster session abstracts, review articles and other types of publications were excluded. Other papers were used for contextualization and discussion. At the end, cases from the involved institution were presented.

## RESULTS

**FIGURE 1:**
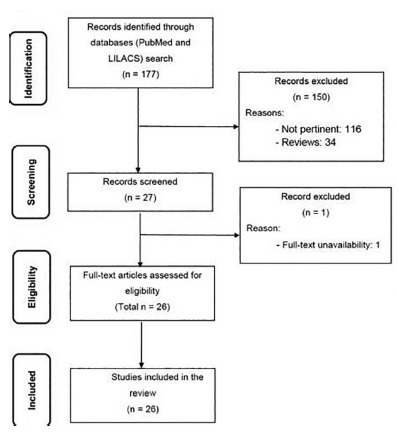
Flow diagram of the review of literature

After extensive online research, 26 studies were included, being 22 case reports and four case series. Table 1 summarizes the main articles found and their reported outcomes. A flow diagram of the review is presented in Figure 1.

**TABLE 1:** Reported cases of pancreaticopleural fistulas secondary to chronic pancreatitis over the last 20 years

Author	Gender/Age (years)	Pancreatitis’ etiology	Presenting symptoms	Imaging diagnosis	Treatment	Outcome
Molinuevo et al.^9^	M/28	Alcoholic	Dyspnea	CT, ERCP	Pleural drainage, Puestow procedure	Asymptomatic after 12 months
	M/37	Alcoholic	Dyspnea	CT, ERCP	Thoracocentesis, Puestow procedure	Asymptomatic after 24 months
	M/41	Alcoholic	Dyspnea	CT, ERCP	Thoracocentesis, Distal pancreatectomy with Roux-en-Y pancreatojejunostomy	Asymptomatic after 20 months
Materne et al.^10^	M/50	Alcoholic	Dyspnea, chest pain	CT, ERCP, MRI	Thoracocentesis, total parenteral nutrition, somatostatin infusion, Distal pancreatectomy with longitudinal pancreatojejunostomy	NR
	M/32	Alcoholic	Dyspnea	CT, ERCP, MRI	Thoracocentesis, somatostatin infusion, placement of an endoscopic stent	Uneventful in the immediate post-procedure period
Neher et al.^11^	M/53	Alcoholic	Dyspnea, chest pain, coughing	CT, ERCP	Thoracocentesis, placement of an endoscopic stent	Without pleural effusion after five months
Takeo et al.^12^	M/67	Alcoholic	NR	CT, ERCP	Thoracocentesis, total parenteral nutrition, octreotide	Asymptomatic at discharge
Ito et al.^13^	M/52	Alcoholic	Coughing, back pain	CT	Pleural drainage, octreotide	NR
	F/39	Alcoholic	Coughing, sputum	CT	Conservative	NR
Akahane et al.^14^	M/69	Alcoholic	Coughing, dyspnea, chest pain	CT, MRI, ERCP	Thoracocentesis, total parenteral nutrition, octreotide	Asymptomatic after five years
Lanternier et al.^15^	M/64	Alcoholic	Dyspnea, coughing, cardiac tamponade	CT, MRI, ERCP	Pleural drainage, placement of endoscopic stent, distal pancreatectomy with pancreatojejunostomy	Without reccurrence after 12 months
Lamme et al.^16^	F / 44	Alcoholic	Dyspnea, coughing	NR	Pleural drainage	Death by pneumonia
	M/54	Alcoholic	Dyspnea, coughing	NR	Pancreatic resection	Recovery in the immediate postoperative period
	M/42	Alcoholic	Dyspnea, coughing	NR	Pancreatic resection	Recovery in the immediate postoperative period
Meybeck et al.^17^	M/39	Alcoholic	Dyspnea, chest pain	CT	Pleural drainage, ocreotide, antibiotics, pleural decortication, percutaneous drainage of pancreatic pseudocyst	Partial involution of the pseudocyst and regression of pulmonary images after six months
Neumann et al.^18^	M/68	NR	Dyspnea, chest pain	CT, ERCP	Placement of an endoscopic stent, antibiotics	Regression after three weeks
Dhebri et al.^5^	F/47	Alcoholic	Dyspnea, chest pain, coughing	CT, ERCP	Pleural drainage, endoscopic sphyncterotomy, octreotide	Lost to follow-up after discharge
	M/46	Alcoholic	Dyspnea, chest pain, coughing	CT, ERCP	Pleural drainage, endoscopic placement of pancreatic stents, octreotide	Doing well after two months
	M/54	Alcoholic	Dyspnea, chest pain	CT, ERCP, MRI	Pleural drainage, ocreotide	Doing well after six months
Zubiaurre et al.^19^	M/40	Alcoholic	Dyspnea, back pain	CT, MRI	Pleural drainage, total parenteral nutrition	Remission after one month
Koshitani et al.^20^	M / 45	Alcoholic	Fever, coughing	CT, ERCP	Pleural drainage, placement of an endoscopic stent	No recurrence after 33 months
	M/56	Alcoholic	Dyspnea	CT, ERCP	Pleural drainage, placement of an endoscopic stent, percutaneous drainage of pancreatic pseudocyst, distal pancreatectomy	No recurrence after 20 months
	M/65	Alcoholic	Dyspnea on exertion	CT, ERCP	Pleural drainage, endoscopic placement of stent	No recurrence after eight months
Cocieru et al.^21^	M/59	Alcoholic	Dyspnea	MRI, ERCP	Thoracocentesis, Frey procedure	No recurrence after three years
Vyas et al. ^22^	M/53	NR	Dyspnea, fever, hemoptysis, chest pain	CT, MRI, ERCP	Pleural drainage, pancreaticojejunostomy	NR
Cooper et al.^23^	M/72	Pancreas pseudodivisum	Dyspnea	CT, EUS, ERCP	Thoracocentesis, EUS-guided placement of pancreatic stent,	No recurrence after one year
Thyagaraj et al.^24^	M/49	Alcoholic, incomplete pancreas divisum	Dyspnea, chest pain, weight loss	CT, MRI	Pleural drainage, distal pancreatectomy	NR
Ferris et al. ^25^	F/51	Alcoholic	Dyspnea, epigastric pain	CT, MRI, ERCP	Thoracocentesis, Endoscopic placement of stent, antibiotics	Resolution in the immediate post-procedure period
Sonoda et al.^26^	M/53	Alcoholic	Dry coughing, dyspnea, heart palpitations	CT, MRI, ERCP	Pleural drainage, total parenteral nutrition, octreotide, distal pancreatectomy	Doing well immediately after recovery from surgery
Shah et al. ^27^	M/32	Alcoholic	Dyspnea, chest pain, coughing, abdominal pain	CT, MRI, ERCP	Endoscopic placement of stent	No recurrence after one year
Mota et al. ^28^	F/52	Alcoholic	Dyspnea	CT	Thoracocentesis, Partingon-Rochelle procedure	Doing well immediately after recovery from surgery
Gomes et al.^29^	M/44	Alcoholic	Dyspnea at exertion, dry coughing, chest pain	CT, ERCP	Thoracocentesis, total parenteral nutrition	Regression of pleural effusion on discharge
Hirosawa et al.^30^	M/58	Alcoholic	Chest pain	CT, ERCP	Pleural drainage, endoscopic placement of stent, antibiotics	Regression of pleural effusion on discharge; patient lost to follow-up and died after two years of an unknown cause
Oh et al.^31^	M/32	Alcoholic	Epigastric pain	ERCP	Pleural drainage, total parenteral nutrition, endoscopic placement of stent	Regression after 4 weeks
	F/47	Post-ERCP Pancreatic duct stricture	Epigastric pain, dyspnea	ERCP, CT, MRI	Endoscopic placement of stent	No recurrence after two months
Sánchez et al.^32^	M/51	Alcoholic	Dyspnea, chest pain	CT, MRI, ERCP	Thoracocentesis, distal enteral nutrition, octreotide, endoscopic placement of stent	Asymptomatic after two years
Soares et al.^33^	M/43	NR (HIV-positive under anti-retroviral therapy)	Dyspnea	CT, MRI, ERCP	Pleural drainage, total parenteral nutrition, somatostatin analogs, endoscopic sphyncterotomy, distal pancreatectomy with Roux-en-Y pancreaticojejunostomy	Regression of pleural effusion 10 days after surgery

M=male; F=female; NR=not reported; CT=computed tomography; MRI= magnetic resonance imaging; ERCP=endoscopic retrograde cholangiopancreatography

Here are added, to the total amount of cases related in the literature over 20 years period, two cases of pancreaticopleural fistula attended in authors institutions, based on retrospective analysis of data collected on medical records. With this addition to total of patients in the literature with this two is 40.

The first case from the authors was related to a 46-year-old man with a history of alcoholism and long-term smoking, admitted to the emergency department due to mild dyspnea, with a diagnosis of right-sided pleural effusion. After a thoracocentesis, an amylase levelof 61,000 IU/l was found in the pleural fluid. Abdominal tomography showed pancreatic changes compatible with chronic pancreatitis. Treatment with oral fasting, total parenteral nutrition, symptomatic medications and thoracocentesis was warranted. Due to the maintenance of a pleural effusion with septa, he underwent a pleural drainage and pleuroscopy. He evolved with high output drainage, and octreotide infusion was indicated. After three weeks of treatment and maintenance of pleural effusion, the patient was referred for an endoscopic retrograde cholangiopancreatography, which showed a dilated and winding main pancreatic duct, with a cranial fistulous pathway, and bleeding externalized by the duodenal papilla, which precluded the placement of a pancreatic stent. He underwent a selective arteriography of celiac trunk and a scintigraphy with marked red cells, both negative for active bleeding. It was opted for the Roux-en-Y pancreaticojejunostomy associated with partial resection of the pancreatic head (Frey procedure). He presented a satisfactory postoperative evolution, with regression of pleural effusion after five days and hospital discharge seven days after surgery. A complete remission of pleural effusion was achieved after two weeks. After 14 months of surgery, he was still was in good conditions with no pain or steatorrhea, and without evidence of endocrine dysfunction.

Another case referred is one 42-year-old male smoker and heavy drinker sought emergency care with a complaint of dyspnea and chest pain for one month. A left-sided pleural effusion was found and a thoracocentesis was performed, showing an amylase level of 250,000 IU/l. An abdominal tomography was performed, which showed changes suggestive of chronic pancreatitis. Endoscopic retrograde cholangiopancreatography showed dilatation and diffuse irregularities of the pancreatic duct and two areas of strictures (head and head-to-body transition) and contrast overflow with formation of a fistulous pathway. A sphyncterotomy and dilation was performed, but the attempt to place a stent was unsuccessful. Surgical treatment was warranted, and a Frey procedure was carried out. He presented a satisfactory postoperative evolution, with regression of the pleural effusion after eight days and hospital discharge the following day. He is currently in the ninth postoperative year of follow-up, using pancreatic enzymes due to exocrine insufficiency, with no pain and no signs of endocrine insufficiency.

## DISCUSSION

Pancreaticopleural fistula is an infrequent complication that may be secondary to acute or chronic pancreatitis, as well as to external or iatrogenic pancreatic trauma. However, this complication is related to chronic pancreatitis of alcoholic origin in 99% of cases^10^.

The pathophysiology of the pancreaticopleural fistula consists of the formation of a posterior pathway of the pancreatic duct to the pleura or, more frequently, after the formation of a pseudocyst and subsequent communication with the pleural cavity. In both cases, the fluid flows through the retroperitoneum through the plane of least resistance into the pleural cavity, usually through the esophageal hiatus. Communications with the pericardium, bronchial tree and esophagus have also been described. Transdiaphragmatic communication is the less common situation^34^ ^,^ ^18^.

Regarding the clinical presentation, Uchiyama et al.^34^ observed that dyspnea, abdominal pain, cough and chest pain are present in 68% of cases. Many patients undergo extensive lung investigation before the pancreas is identified as the primary site of the disease. Abdominal symptoms are infrequent. Pancreatic ascites are associated with pancreaticopleural fistula in 20% of cases, and in 4% there is an association with pericarditis^5^.

Diagnosis is usually performed by thoracocentesis after chest radiography, with laboratory findings of elevated levels of amylase and lipase in the pleural fluid. Serum amylase has no diagnostic validity, since it is low in some cases^5^ ^-^ ^9^. The differential diagnosis of pleural effusions should be made with acute pancreatitis, gynecological, pulmonary, and metastatic tumors, pneumonia, esophageal perforation, lymphoma, leukemia and pulmonary tuberculosis^13^ ^,^ ^14^ ^,^ ^7^ ^,^ ^19^ ^,^ ^23^ ^,^ ^32^ ^,^ ^12^ ^,1,^ ^17^ ^,^ ^16^. The diagnosis can be confirmed by endoscopic retrograde cholangiopancreatography in 80% of the cases, showing the fistulous pathway in 59%. In 70% of cases, computed tomography associated with it identifies the fistulous path. Magnetic resonance cholangiopancreatography may demonstrate pancreatic involvement and fistula, without the need for contrast, constituting a non-invasive alternative^34^ ^,^ ^5^ ^,^ ^20^ ^,^ ^24^ ^,^ ^36^ ^,^ ^15^ ^,^ ^3^ ^,^ ^35^.

There are no randomized studies that indicate the most appropriate treatment of pancreaticopleural fistulas. At first, clinical management with parenteral nutrition and infusion of somatostatin analogs are performed for two to three weeks, with or without pleural drainage. However, resolution of the anatomical continuity of the pancreatic duct is what defines the good evolution of the condition. The efficacy of conservative treatment varies from 30-60% in some series and from 0-33% in others^5^. Recently, endoscopic treatment has been more widely performed, consisting of balloon dilatation and placement of intraductal prostheses, with success rates of up to 25% being reported with this treatment modality^18^ ^,^ ^5^ ^,^ ^23^ ^,^ ^24^ ^,4,^ ^33^ ^,^ ^6^ ^,^ ^30^ ^,^ ^28^ ^,^ ^26^ ^,^ ^27^.

The main indication for surgical treatment is the failure of clinical and/or endoscopic treatments^34^ ^,^ ^18^ ^,^ ^5^ ^,^ ^29^. Surgery is based on internal pancreatic drainage, especially by means of pancreaticojejunostomy, and/or pancreatic resections, depending on the degree of involvement of the main duct and the pancreatic portion involved. A review by King et al.^14^ observed that attempts at prolonged periods of medical therapy tend to delay the resolution of the fistula compared with patients who undergo definitive operative intervention early in the course of treatment.

There is no consensus regarding the optimal treatment. Conservative management should be the first option; despite its low rates of complete resolution, there are reports of success and this modality avoids the possibility of complications arising from invasive procedures; however, it is often associated with lengthier hospital stays^32^ ^,^ ^12^ ^,1,^ ^36^ ^,6,^ ^22^ ^,^ ^11^. Endoscopic treatment should be the second-line therapy, indicated for those individuals who did not respond to clinical measures, since it presents good results and lower morbidity and mortality than surgery^5^ ^,^ ^23^ ^,^ ^24^ ^,4,^ ^28^ ^,^ ^26^ ^,^ ^27^. Hence, surgery should be warranted in the refractory cases^21^ ^,^ ^19^ ^,^ ^17^ ^,^ ^15^ ^,^ ^3^ ^,^ ^35^ ^,^ ^33^ ^,^ ^30^ ^,^ ^9^ ^,^ ^29^. There is no consensus in regard to the optimal technique to be adopted; it must depend on the individual characteristics of each case. Individuals with predominantly cephalic disease would benefit from Frey or Beger procedures^3^; those with diffuse dilatation of the duct without severe involvement of the pancreas head would be appropriately treated by means of a Puestow/Partington-Rochelle procedure ^9^ ^,^ ^15^ ^,^ ^22^ ^,^ ^28^; those with disease restricted to the pancreas tail or distal body would benefit from distal pancreatectomies, with or without pancreaticojejunostomy, depending on the caliber of the pancreatic duct^16^ ^,^ ^15^ ^,^ ^33^ ^,^ ^30^. Since surgery is reportedly the best treatment approach to treat the abdominal symptoms, especially refractory pain^8^ ^,^ ^7^ ^,^ ^2^ ^,^ ^31^ ^,^ ^25^, it should also be considered a more definitive treatment for these individuals, since it may bring a more integrative relief of both thoracic and abdominal consequences of the disease.

## CONCLUSION

Pancreaticopleural fistula is a rare complication of chronic pancreatitis and the Frey procedure may be an appropriate treatment option when clinical and endoscopic treatments are unsuccessful.
